# Refining the medical student safety attitudes and professionalism survey (MSSAPS): adaptation and assessment of patient safety perception of French medical residents

**DOI:** 10.1186/s12909-019-1667-y

**Published:** 2019-06-21

**Authors:** Stéphanie Larramendy-Magnin, Emmanuelle Anthoine, Barbara L’Heude, Brice Leclère, Leïla Moret

**Affiliations:** 1 Department of General Practice, Faculty of Medicine, University of Nantes, Nantes, France; 2 Department of Public Health, Nantes University Hospital, 85 rue Saint Jacques 44093 Nantes Cedex 1, Nantes, France; 3 UMR 1246 INSERM SPHERE “MethodS in Patients-centered outcomes and HEalth ResEarch”, Universities of Nantes and Tours, Nantes, France; 4 Basse-Goulaine, Nantes, France; 5 Department of Public Health, Nantes University Hospital, Nantes, France

## Abstract

**Background:**

Implementing a patient safety curriculum for medical students requires to identify their needs and current awareness of the topic. Several tools have been developed to measure patient safety culture, but none of them have been developed in the French context.

Our objective was to adapt and refine the psychometric properties of the MSSAPS, developed by Liao et al, to use it among general practice (GP) residents.

**Methods:**

1-We conducted a translation and transcultural adaptation of the MSSAPS questionnaire (28 items, 5 dimensions: safety culture, teamwork culture, experiences with professionalism, error disclosure culture and comfort expressing professional concerns) in accordance with the international recommendations.

2-We studied the new questionnaire’ psychometric properties on a sample of GP residency students in 2016. This validation comprised 2 steps: a confirmatory factor analysis (CFA) for each dimension of the MSAPPS to explore the adequacy of the structure of the questionnaire; an exploratory factor analysis to refine the instrument, using a principal component analysis and Cronbach’s α-coefficients calculation. A final CFA examined the structure validity of the refined questionnaire.

3-We described the items and the safety cultural scores in our sample of residents.

**Results:**

Among 391 eligible students, 213 responded (54%).

The initial structure was not confirmed by CFAs, showing a poor fit for 3 of the 5 dimensions: safety culture, teamwork culture and professionalism. Exploratory PCA led to 3 dimensions: Safety culture (PVE: 18.5% and 7 of 8 initial items), Experiences with professionalism (PVE: 17.8% and 5 of 7 initial items) and Error disclosure culture (PVE: 13.6% and 3 of 4 original items). Cronbach’s α-coefficients were 0.74, 0.78 and 0.76 respectively. The final CFA confirmed the existence of the 3 latent dimensions with a good fit to the and highly significant structural coefficients (*P* < 0.001). Mean scores were equal to 65.4 [63.6; 67.6] for the safety culture, 66.9 [63.8; 70.1] for the experience with professionalism, and 54.4 [51.6; 57.2] for the error disclosure culture.

**Conclusion:**

This study reports satisfactory psychometrics properties of the French version of the MSAPPS and provides evidence of important training needs for GP residents in the field of patient safety culture.

## Background

Since the publication in 2000 of the alarming report “To err is human” by the Institute of Medicine in the USA, reducing the prevalence and severity of medical errors has become a priority for hospitals all around the world [1, 2]. Among the levers of improvement, developing patient safety culture could help healthcare professionals to consider patient safety as a constant concern. Patient safety culture has been defined by The European Society for Quality in Health Care as “an integrated pattern of individual and organizational behavior, based upon shared beliefs and values, that continuously seeks to minimize patient harm which may result from the processes of care delivery” [3]. This functional and open definition shows that patient safety culture does not only concern healthcare professionals but also students, who play a key role in enhancing patient safety in hospitals. But even though patient safety culture has undoubtedly improved among healthcare professionals in the last decade, it is still underdeveloped in medical students’ curricula [4, 5], despite the guide published in 2008 by the World Alliance for Patient Safety to develop patient safety curriculum in medical schools [6, 7].

The first step to implement a patient safety curriculum for medical students is to identify their needs and current awareness of the topic. Several tools have been developed to measure patient safety culture [8–10]. The most popular self-administered questionnaire for measuring PSC in hospitals, the “Hospital Survey On Patient Safety Culture” questionnaire (HSOPSC), is dedicated to healthcare professionals and does not completely apply to residents, because they are not fully integrated in care units and hospital decisions [8]. A recent literature review identified several useful tools for assessing PSC of medical residents, but none of them related to PSC within hospitals, which is where residents do most of their training [9]. From several existing questionnaires (Safety Attitudes Questionnaire-SAQ [10] and HSOPSC), Liao et al developed and validated The Medical Student Safety Attitudes and Professionalism Survey (MSSAPS), a questionnaire that focuses on promoting curricular and cultural change for residents during their hospital rotations [11]. However, several aspects of its psychometric validation could be improved: some dimensions had unsatisfactory fit indices, one of them was saturated, and the fit of the overall model was not reported.

Our objective was to perform a transcultural adaptation of the American version of the MSSAPS and to validate the French version (MSSAPS-F) by refining its psychometric properties. We used the MSSAPS-F to evaluate the patient safety culture of general practice (GP) residents of the University of Nantes in order to guide the development of their educational and behavioral support.

## Methods

### Translation and transcultural adaptation

The MSSAPS’s questionnaire includes 28 items measuring 5 dimensions: safety culture, teamwork culture, experiences with professionalism, error disclosure culture and comfort expressing professional concerns [11].

The translation and transcultural adaptation of the questionnaire were conducted in accordance with the international recommendations [12]. An expert panel composed of four native French speakers (a general practitioner, a public health practitioner, a general medicine resident and a nonmedical researcher) independently translated the questionnaire in French. Discrepancies between the translations were discussed in order to produce a unique reconciled version. This reconciled French version was then back-translated into English by two English native-speaking translators, who were blinded to the original American version. The back-translated and original versions of the MSSAPS were then compared to make sure that the original meaning of the items was preserved and to adjust the French version if needed. To enhance face validity, this French version was tested on a panel of 10 students, whereupon three items were reformulated for better clarity.

### Participants and survey administration

The survey was carried out from March to June 2016 at the Faculty of medicine of Nantes University, France. Every student of the 3-year GP residency was invited to participate. The participants were included during a pedagogical meeting on March 26th. The consenting residents were asked to fulfill a paper questionnaire, which consisted of the French version of the MSSAPS items and additional questions about their gender, age, and year of residency. Residents who could not attend the meeting were invited to participate via an e-mail, which linked to an online version of the questionnaire. The e-mail was sent in April, and was followed by two reminders in May and June.

### Statistical analysis

#### Psychometric validation process

As in the original article [11], the first step of the psychometric analysis consisted in five confirmatory factor analyses (CFA), one for each dimension. In addition, we performed two CFAs on the whole scale: one estimating covariances between the dimensions, the other one hypothesizing that the dimensions were independent.

As these analyses revealed that the adequacy of the structure was not sufficient, we also performed an exploratory factor analysis (EFA). The items with a rate of non-available values (missing values and “Not Applicable” answers) superior to 20%, or a floor/ceiling effect superior to 50% were eliminated. If two items exhibited a Pearson’s correlation coefficient superior to 0.6, only the more relevant of the two was kept.

With the remaining items, we tried to identify the underlying dimensions using a principal component analysis (PCA) with a Varimax rotation. For an item to be attributed to a dimension, the corresponding factor loading should be superior to 0.40. When an item exhibited factor loading superior to 0.40 for several dimensions, it was attributed to the one for which the internal consistency (Cronbach’s α-coefficient) was maximized [13]. Overall, Cronbach’s α-coefficients superior or equal to 0.70 were regarded as satisfactory [13].

A final CFA was performed to examine the structure validity of the refined questionnaire. The model hypothesized from the exploratory analysis was tested to confirm how well the data fitted the postulated structure [14]. The following fit indices were specifically considered: the comparative fit index (CFI), the Tucker-Lewis index (TLI), the incremental fit index (IFI), the standardized root mean residual (SRMR) and the root mean square error approximation (RMSEA). CFI, TLI and IFI values above 0.90 and RMSEA and SRMR values under 0.08 were indications of a good fit [14].

All the statistical analyses were done using R version 3.4.3.

#### Score calculation

Item responses were rated using five-level Likert scales (from 1 = ‘stronly disagree’ to 5 = ‘strongly agree’), with the addition of a ‘Not Applicable’ modality. As we wanted to calculate individual scores for each dimension of the scale, the typical way to report culture scores described by the Agency for Healthcare Research and Quality [8] was not appropriate. Instead, we used the method suggested by Sexton [10, 15]: individual scores corresponded to the mean of the individual response levels for all the items, minus 1 and multiplied by 25 so that the score ranged from 0 to 100 (the levels of the negatively-worded items were reversed). The mean score for a dimension was the sum of the individual scores divided by the number of respondents.

#### Descriptive statistics

The quantitative variables were described using their mean ± standard deviation and the qualitative variables were described using counts and proportions (%).

## Results

### Characteristics of the sample

A total of 391 students were eligible to participate in the study, 244 of them attended the initial meeting. The majority of the questionnaires (*n* = 203) were completed during the meeting and 10 of them were completed online. The participation rate varied from 67% for first-year residents to 51 and 46% for second-year and third-year residents respectively. The overall participation rate was 54% (*n* = 213). The mean age of the respondents was 26 ± 1.4 years (range: 23–31 years), and two thirds of the respondents were women (66.5%). Most of the respondents were in their first year of residency (57%), a quarter of them were in their second year (27%) and a third in their final year (34%).

### MSSAPS-F questionnaire psychometric validation

#### First step: test of the original structure

The dimension-specific CFAs showed a poor fit to the hypothesized structure: the fit indices were not satisfactory for 3 of the 5 dimensions: safety culture, teamwork culture and professionalism. The dimension called `comfort expressing professional concerns` required six values to be estimated (a coefficient/error pair for each of the 3 items) based on a variance-covariance matrix that also consisted of six unique values (3 variances and 3 pairwise covariances). This situation corresponds to a just-identified model, for which the fit indices are trivially perfect but not interpretable. Unsurprisingly, the fit indices for the two whole-scale CFAs were not satisfactory either (Table 1).

**Table 1:** Confirmatory factor analyses of the original version of the MSSAPS

Factor	No of items	Df	RMSEA	SRMR	CFI	TLI	IFI
Safety culture	8	20	0.074	0.055	0.917	0.883	0.919
Teamwork culture	6	9	0.139	0.060	0.885	0.808	0.888
Error disclosure culture	4	2	0.0	0.019	1.0	1.012	1.004
Professionalism	7	14	0.111	0.060	0.915	0.872	0.916
Comfort expressing professional concerns	3	0	0.0	0.0	1.0	1.0	1.0
Global CFA with 5 dependent factors	28	340	0.058	0.076	0.848	0.831	0.853
Global CFA with 5 independent factors	28	350	0.077	0.175	0.729	0.707	0.736

#### Second step: refinement of the original structure

Among the 28 items, 2 were removed because of a floor effect (“I felt that a patient was discriminated against by a member of my team on the basis of gender, race, sexual orientation or religion” and “I have received education or training on how to disclose medical errors to patients”) and 3 because of a ceiling effect (“The quality of care received by patients was impacted by teamwork”, “I had good collaboration with nurses” and “I had good collaboration with team members”) (Table 2).

**Table 2:** Distribution of residents’ responses on items of The Medical Student Safety Attitudes and Professionalism Survey (MSSAPS)

Survey items by cultural factor	Agree strongly		Agree sslightly		Neutral		Disagree slithly		Disagree strongly		Not Applicable	
	*n*	%	*n*	%	*n*	%	*n*	%	*n*	%	*n*	%
Safety Culture												
I received appropriate feedback about my performance	44	**21.0%**	111	**52.9%**	28	**13.3%**	25	**11.9%**	2	**1.0%**	3	**1.4%**
We followed standard operating procedures guidelines and protocols for the floor	47	**24.0%**	79	**40.3%**	17	**8.7%**	40	**20.4%**	13	**6.6%**	17	**8.0%**
I observed excellent patient safety practices	25	**12.3%**	82	**40.2%**	66	**32.4%**	27	**13.2%**	4	**2.0%**	9	**4.2%**
Medical errors were handled appropriately	31	**15.5%**	95	**47.5%**	59	**29.5%**	13	**6.5%**	2	**1.0%**	13	**6.1%**
I was encouraged by colleagues to report any patient safety concerns I may have had	33	**16.3%**	66	**32.7%**	57	**28.2%**	34	**16.8%**	12	**5.9%**	11	**5.2%**
The clinical culture made it easy to learn from the errors of others	58	**28.6%**	93	**45.8%**	27	**13.3%**	21	**10.3%**	4	**2.0%**	10	**4.7%**
I would have felt safe being treated here as a patient	66	**33.0%**	92	**46.0%**	20	**10.0%**	20	**10.0%**	2	**1.0%**	13	**6.1%**
I knew the proper channels to direct questions regarding patient safety	39	**19.0%**	71	**34.6%**	34	**16.6%**	49	**23.9%**	12	**5.9%**	8	**3.8%**
Teamwork Culture												
The quality of care received by patients was impacted by teamwork	114	**58.2%**	70	**35.7%**	8	**4.1%**	3	**1.5%**	1	**0.5%**	17	**8.0%**
I had good collaboration with nurses	135	**75.0%**	37	**20.6%**	8	**4.4%**	0	**0.0%**	0	**0.0%**	33	**15.5%**
I had good collaboration with team members	142	**72.4%**	48	**24.5%**	5	**2.6%**	1	**0.5%**	0	**0.0%**	17	**8.0%**
Disagreements were resolved appropriately	46	**22.8%**	105	**52.0%**	25	**12.4%**	25	**12.4%**	1	**0.5%**	11	**5.2%**
I had the support I needed from other personnel to care for patients	93	**45.8%**	100	**49.3%**	7	**3.4%**	3	**1.5%**	0	**0.0%**	10	**4.7%**
It was easy for personnel to ask questions when there was something that they did not understand	96	**50.5%**	75	**39.5%**	13	**6.8%**	5	**2.6%**	1	**0.5%**	23	**10.8%**
Experiences with professionalism												
A member of my team made disparaging or demeaning remarks about one of our patients	32	**16.2%**	52	**26.3%**	35	**17.7%**	43	**21.7%**	36	**18.2%**	15	**7.0%**
A member of my team was disrespectful to someone below himself/herself on the team ranking	13	**6.8%**	26	**13.7%**	30	**15.8%**	46	**24.2%**	75	**39.5%**	23	**10.8%**
The attending physician answered patients’ questions inadequately or simply ignored them	14	**7.1%**	48	**24.5%**	26	**13.3%**	53	**27.0%**	55	**28.1%**	17	**8.0%**
The resident answered patients’ questions inadequately or simply ignored them	4	**2.1%**	21	**11.1%**	38	**20.1%**	58	**30.7%**	68	**36.0%**	24	**11.3%**
One of my superiors behaved inappropriately, but I did not report it because I was afraid it would affect my evaluation	10	**5.3%**	16	**8.4%**	22	**11.6%**	50	**26.3%**	92	**48.4%**	23	**10.8%**
I felt that a patient was discriminated against by a member of my team on the basis of gender, race, sexual orientation or religion	4	**2.0%**	10	**5.0%**	9	**4.5%**	48	**24.0%**	129	**64.5%**	13	**6.1%**
A member of my team was rude and disrespectful to a patient or family member	6	**3.0%**	21	**10.4%**	12	**6.0%**	62	**30.8%**	100	**49.8%**	12	**5.6%**
Error disclosure Culture												
When errors are made. They are disclosed to patients/families	20	**10.1%**	92	**46.2%**	41	**20.6%**	42	**21.1%**	4	**2.0%**	14	**6.6%**
The culture during my rotations made it easy to disclose medical errors	12	**6.6%**	56	**30.8%**	57	**31.3%**	49	**26.9%**	8	**4.4%**	31	**14.6%**
I am encouraged by my colleagues to disclose errors to patients/families	12	**6.3%**	45	**23.6%**	75	**39.3%**	47	**24.6%**	12	**6.3%**	22	**10.3%**
I have received education or training on how to disclose medical errors to patients	5	**2.5%**	9	**4.4%**	19	**9.4%**	44	**21.7%**	126	**62.1%**	10	**4.7%**
Comfort expressing professional concerns												
I felt comfortable expressing my concerns about patient safety to my superiors	37	**18.6%**	89	**44.7%**	37	**18.6%**	31	**15.6%**	5	**2.5%**	14	**6.6%**
I felt comfortable expressing my concerns about patient treatment to my superiors	63	**31.5%**	87	**43.5%**	17	**8.5%**	25	**12.5%**	8	**4.0%**	13	**6.1%**
I felt comfortable expressing my concerns about my own mistreatment to my superiors	40	**20.4%**	57	**29.1%**	53	**27.0%**	31	**15.8%**	15	**7.7%**	17	**8.0%**

Three pairs of items had Pearson’s correlation coefficients exceeding 0.6, leading to the removal of three items: “I had the support I needed from other personnel to care for patients” and “It was easy for personnel to ask questions when there was something that they did not understand”, “The attending physician answered patients’ questions inadequately or simply ignored them” and “The resident answered patients’ questions inadequately or simply ignored them”, “I felt comfortable expressing my concerns about patient safety to my superiors” and “I felt comfortable expressing my concerns about patient treatment to my superiors”).

Five exploratory PCAs were then performed on the remaining 20 items, which led to the identification of three dimensions. Five items were removed because of their low factor loadings on one of the three factors, or because they exhibited high factor loadings on two of them. No item maximized the Cronbach’s α-coefficient and all the 15 remaining items had factor loadings superior or equal to 0.50 within their own dimension.

MSAPPS-F, composed of 15 items and 3 dimensions is presented in Table 3. The first dimension corresponded to safety culture, and was composed of seven of the eight items of the original dimension. The second one corresponded to experiences with professionalism, and was composed of five of the seven items of the original dimension. The last one corresponded to error disclosure culture, and was composed of three of the four items of the original dimension. These new dimensions accounted for 18.5, 17.8 and 13.6% of the variance respectively. Their respective Cronbach’s α-coefficients were 0.74, 0.78 and 0.76.

**Table 3:** Results of the PCA using Varimax rotation

		Scale dimensions		
Short name	Label (in French and in English)	SC^1^	EP^2^	EDC^3^
SC1	We followed standard operating procedures guidelines and protocols for the floor *Dans le service nous avons suivi des procédures standardisées, des recommandations et des protocoles (par ex: lavage de mains, check-lists pour éviter les infections nosocomiales…)*	**0.68**	0.04	0.08
SC2	I observed excellent patient safety practices *J’ai observé d’excellentes pratiques en matière de sécurité du patient*	**0.65**	0.11	0.09
SC3	Medical errors were handled appropriately *Les erreurs médicales ont été gérées de façon appropriée*	**0.50**	0.02	0.30
SC4	I was encouraged by colleagues to report any patient safety concerns I may have had *J’ai été encouragé par les collègues à signaler toute préoccupation que j’aurais pu avoir quant à la sécurité des soins*	**0.55**	0.09	0.15
SC5	The clinical culture made it easy to learn from the errors of others *Le fonctionnement du service facilitait l’apprentissage à partir des erreurs des autres*	**0.69**	0.13	0.03
SC6	I would have felt safe being treated here as a patient *Je me serais senti en sécurité en tant que patient dans ce service*	**0.61**	0.17	0.03
SC7	I knew the proper channels to direct questions regarding patient safety *Je savais où adresser mes questions concernant la sécurité des patients*	**0.61**	0.07	0.18
EP1	A member of my team made disparaging or demeaning remarks about one of our patients *Un membre de l’équipe a fait des remarques désobligeantes ou dévalorisantes à propos d’un patient*	0.05	**0.76**	0.06
EP2	A member of my team was disrespectful to someone below himself/herself on the team ranking *Un membre de l’équipe a manqué de respect envers un subordonné hiérarchique*	0.03	**0.69**	0.08
EP3	The attending physician answered patients’ questions inadequately or simply ignored them *Un médecin du service a répondu de façon inadéquate aux questions d’un patient ou les a simplement ignorées*	0.12	**0.78**	0.11
EP4	One of my superiors behaved inappropriately, but I did not report it because I was afraid it would affect my evaluation *Un de mes supérieurs s’est comporté de manière inappropriée, mais je ne l’ai pas signalé de peur que cela n’affecte mon évaluation de stage*	0.06	**0.65**	0.13
EP5	A member of my team was rude and disrespectful to a patient or family member *Un membre de l’équipe s’est montré impoli ou irrespectueux envers un patient ou un membre de sa famille*	0.08	**0.69**	0.11
EDC1	When errors are made, they are disclosed to patients/families *Lorsque des erreurs sont commises, les patients et/ou leur famille en sont informés*	0.04	0.01	**0.85**
EDC2	The culture during my rotations made it easy to disclose medical errors *L’état d’esprit dans le service était propice à l’annonce des erreurs médicales*	0.22	0.19	**0.75**
EDC3	I am encouraged by my colleagues to disclose errors to patients/families *Mes collègues m’encourageaient à informer les patients et/ou leur famille des erreurs commises*	0.22	0.16	**0.73**

^1^SC: Safety culture dimension

^2^EP: Experience with professionalism dimension

^3^EDC: Error disclosure culture

Footnote: Factor loadings of the items in each dimension are highlighted in bold

**Fig. 1:**
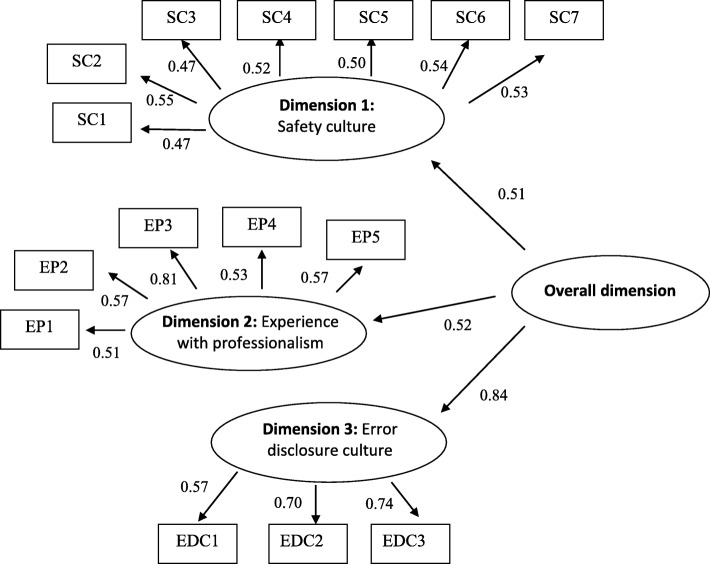
Confirmatory factor analysis of the MSSAPS-F

The CFA confirmed the existence of the 3 latent dimensions and of a global score (Fig. 1). All the indices indicated a good fit to the data (CFI = 0.917; TLI = 0.900; IFI = 0.920; SRMR = 0.070; RMSEA = 0.054) and the structural coefficients were highly significant (*P* < 0.001).

### Description of items and of the three safety cultural scores

The description of the answers to each item is available in Table 2. Mean scores were equal to 65.4 [63.6; 67.6] for the safety culture, 66.9 [63.8; 70.1] for the experience with professionalism, and 54.4 [51.6; 57.2] for the error disclosure culture. For the whole scale, the mean score was equal to 63.9 [62.1; 65.7]. No significant differences were observed between genders, ages and years of residency (Results not shown).

## Discussion

Our study was the first experiment carried out in France that assessed the safety culture of GP residents. It provides descriptive results that can be used to assess and follow the progress of the residents’ perception of safety culture. The French adaptation of the MSSAPS (MSSAPS-F) has been developed in accordance with the standards [16]: the initial instrument was translated and back-translated, and the structure and reliability were analyzed using a sample of 213 students. Using the same methodology as Liao et al in the original study [11], we observed the same validation issues that they encountered: the dimensions labeled ‘safety culture’ and ‘teamwork culture’ did not seem to fit the hypothesized structure well. Additionally, in our analysis, the dimension called ‘comfort expressing professional concerns’ and ‘experiences with professionalism’ had either unsatisfactory or interpretable fit indices. As the specific results for these dimensions are not available in the original study, we could not check if these problems were also encountered by Liao et al [11], but it seems likely at least for the just-identification issue.

To improve the structure, we performed exploratory and confirmatory factor analyses. The result was a questionnaire with very satisfying psychometric properties, and shorter than the original one: it was composed of 15 items and 3 dimensions, whereas the original one had 28 items and 5 dimensions. Two dimensions were completely removed (teamwork culture and comfort expressing professional concerns) and three were shortened (safety culture, error disclosure culture, and experiences with professionalism).

We believe that the remaining three dimensions are indeed the most relevant for assessing patient safety culture: the ‘safety culture’ dimension explores several domains of safety culture (protocols and care procedures, non-punitive response to errors, organizational learning), the ‘error disclosure culture’ dimension has been shown to predict intent to disclose a error better than other measures of healthcare culture [17], and the ‘experiences with professionalism’ dimension corresponds to what Hafferty called the “hidden curriculum” [18] (i.e. all the messages transmitted implicitly through everyday vocabulary, practices and habits [19]).

While teamwork culture is a well-known dimension of patient safety culture [8, 20], its evaluation might not be as relevant for residents. Indeed, as they only work there for a few months, medical students and residents are not fully integrated in the care units. Incidentally, for them, the notion of unit relates to a common core of academic training, rather than to a group work in a care unit [9]. Finally, while the three items of the dimension labeled ‘comfort expressing professional concerns’ addressed interesting issues, they seemed to be very similar to some items of the other dimensions (e.g. “medical errors were handled appropriately” or “I am encouraged by my colleagues to disclose errors to patients/families”).

Additional work is however needed to confirm the psychometric properties of our refined scale. Indeed, our evaluation was based on a voluntary sample with a rather low participation rate, which might not be representative of our target population. This potential source of bias, however, is pretty common, and the original MSSAPS study had a response rate close to the one we observed [11]. It would be interesting, for external validity, to test if our abridged version could improve the fit indices of the original study. Several other aspects of the MSSAPS-F could also be questioned. For example, the fact that the items of the dimension ‘experiences with professionalism’ are worded negatively, whereas all the other ones are worded positively might influence how the students answer to this specific dimension. The choice of a five-level Likert scale could also be questioned. Indeed, in Likert scales with an odd number of response categories, the middle category can be seen as a neutral option that respondents can take when they are not sure what to answer. For the purpose of safety culture evaluation, it might be more relevant to force the respondents to make a more informative choice using an even number of categories.

However, this neutral response category can be interesting for improving the residents’ patient safety curriculum. For example, a lot of items of the ‘safety culture’ dimension had a high rate of neutral answers. This result suggested that efforts had to be made to raise students’ awareness about patient safety in healthcare. Currently, indeed, the mandatory formal training on this matter for French medical students is very limited and often targeted at healthcare-acquired infections.

For the ‘experiences with professionalism’ dimension, the residents’ mean score was 66.9/100, underlining the critical perception that residents have about their superiors and the healthcare teams. These results question the potential influence of some inadequate behaviors of team members on the residents’ behaviors—the negative side of the hidden curriculum. It has been shown that teams lacking professionalism have a negative impact on the residents [21].

The lowest score was observed for the ‘error disclosure culture’ dimension (54.4/100). One particularly remarkable result of this dimension is that the large majority of the residents (84.1%) declared that they had never received any sort of training on how to disclose medical errors to patients. Unfortunately, as a general rule, residents’ superiors often cannot be regarded as models on this particular issue, given their own lack of training [19]. This situation can lead residents to minimize or even deny medical errors, which in turn may lead to an under-reporting of adverse events [22], and to defensive medicine practices [23–25].

In this context, the development and implementation of a specific training pack devoted to residents are highly needed, and must be complemented by continuous education packs for the supervisors. These packs should contain, among others, formal lectures, trainings, problem-solving learning and psychological support [26, 27] to make the students more comfortable with identifying, analyzing and disclosing adverse events.

Following this first overview, the training of the GP residents at the University of Nantes was modified in several ways. Every two months, under supervision, first-year residents are now involved in experience sharing groups, that help them discuss and analyze medical errors that might have occurred during their rotations. A psychological support group is also available for residents who might have problems coping with bad experiences. Moreover, the first-year residents receive a theoretical training complemented by case reports during a one-day seminar on medical errors analysis and error disclosure to patients. In addition, an e-learning program based on the WHO curriculum guide was developed and has become mandatory for all GP residents^27^ in their first year. In terms of follow-up indicators, the MSSAPS-F questionnaire is now completed at the beginning of each year of residency and will be helpful to evaluate the practicum sites.

## Conclusions

This first study on residents’ patient safety culture carried out in France provides evidence of important training needs for GP residents in the field of patient safety culture. The transcultural adaptation and validation of the MSSAPS questionnaire resulted in a shorter and more relevant tool. A larger experiment in a panel of French universities could help to validate this tool and develop improvement actions at a national level.

## Data Availability

The datasets used and analysed during the current study are available from the corresponding author on reasonable request.

## Abbreviations

CFA: Confirmatory Factor Analysis
CFI: The Comparative Fit Index
EFA: Exploratory Factor Analysis
GP: General Practice
IFI: The Incremental Fit Index
MSSAPS: The Medical Student Safety Attitudes and Professionalism Survey
PCA: Principal Component Analysis
RMSEA: The Root Mean Square Error Approximation
SRMR: The Standardized Root Mean Residual
TLI: The Tucker-Lewis Index
